# Conformations of the Pyranoid Sugars. III. Infrared Absorption Spectra of Some Acetylated Aldopyranosides

**DOI:** 10.6028/jres.064A.042

**Published:** 1960-10-01

**Authors:** R. Stuart Tipson, Horace S. Isbell

## Abstract

The infrared absorption spectra of twenty-four acetylated aldopyranosides in the range of 5,000 to 250 cm^−1^ are reported. The conformation adopted by each of seventeen of the corresponding *unacetylated* glycosides had previously been assigned by us from a study of their infrared spectra. Analysis of the spectra revealed, for the acetylated glycosides (as for the parent glycosides), groups of absorption bands which showed a concerted shift on change of anomeric disposition. Assignment of conformation by the methods developed earlier led to the conclusion that each acetylated glycoside has the same conformation as its parent glycoside.

Intercomparison of the spectra of four of the remaining acetates with those of related acetates, especially in regard to the characteristic groups of absorption bands, afforded evidence that the anomeric methoxyl group is axial in methyl hepta-*O*-acetyl-4-*O-β*-d- glucopyranosyl-*α*-d-mannopyranoside, and equatorial in methyl tetra-*O*-acetyl-*β*-d-manno pyranoside, methyl penta-*O*-acetyl-d-*glycero-β*-l-manno-heptopyranoside, and methyl hepta- *O*-acetyl-4-*O-β*-d-glucopyranosyl-*β*-d-mannopyranoside. For lack of spectra of related acetylated aldopyranosides, assignments cannot yet be made for methyl hepta-*O*-acetyl- 6-*O-β*-d-glucopyranosyl-*β*-d-glucopyranoside, methyl penta-*O*-acety-d-*glycero-β*-d-*ido*-heptopyranoside, and methyl hepta-*O*-acetyl-4-*O-β*-d-galactopyranosyl-*β*-d-altropyranoside.

## 1. Scope and Purpose of the Project

The purpose of the present project was twofold. The first objective was to record the infrared absorption spectra of a variety of acetylated aldopyranosides for use in the identification of supposedly identical samples. The spectra of three of the compounds included in the present study were recorded by Kuhn [1]^1^ for a limited range (1,250 to 667 cm^−1^). In the following year, the spectra of 13 of the compounds were given (for the range of 5,000 to 667 cm^−1^) by Isbell and coworkers in a privately circulated report [2] which was subsequently published 13]. Twelve of the spectra obtained by these investigators were later discussed by Whistler and House [4], who noted certain bands in the range of 1,205 to 855 cm^−1^. Next, Barker and coworkers [5] examined the spectra of two of Kuhn’s compounds and of four others that are included in the present study, but have not published them in sufficient detail to permit comparison over a wide spectral range; some of the bands in the range of 960 to 730 cm^−1^ were discussed. In the present article, the infrared absorption spectra of 24 acetylated aldopyranosides, in the range of 5,000 to 250 cm^−1^, are given; all of the bands of all of these compounds have been measured and have received consideration.

The second objective was the discovery of correlations that might be of value in structural analysis, both as regards (a) the presence of certain functional groups and (b) the particular conformation assumed by each compound. Isbell and coworkers [3] recorded the infrared spectra of 13 of the compounds dissolved in suitable solvents, and were able to reach certain conclusions regarding correlations of the kind mentioned. However, at the low concentrations they employed, minor bands were absent or difficult to detect. The spectrograms have now been recorded for the solid phase, as pellets consisting of crystalline material suspended in an alkali-metal halide. [Such spectra show more bands than those for the same compounds dissolved in a solvent. This is because, for the solid, the following factors are operative: (1) A removal of degeneracies by perturbations from lattice forces; (2) occurrence of combination vibrations between molecular and lattice modes in the solid; and (3) an intensification of overtones and combination vibrations.]

In part II of this series of articles [6], a method was described for gaining information regarding the conformations of unsubstituted aldopyranosides from analysis of their infrared spectra. The analysis revealed groups of absorption bands, characteristic for each group-configuration, which displayed a concerted shift on change of anomeric disposition. This empirical observation was employed, in conjunction with a consideration of instability factors (arrived at on theoretical grounds), in making conformational assignments and in deciding the arrangement of groups (e.g., axial or equatorial) at the anomeric carbon atom of these and related compounds. The results were essentially in agreement with those obtained experimentally by Reeves [7] from a study of the behavior of these unsubstituted aldopyranosides on forming complexes with the cuprammonium reagent. The present article describes the results obtained on applying the same kind of analysis to the infrared spectra of the fully acetylated derivatives of 17 of the alclopyranosides studied in part II and of 7 acetylated aldopyranosides whose unacetylated parents were not included therein.

## 2. Compounds Investigated

Table 1 gives a list of the compounds, their code numbers [8], and an index to the spectrograms; the serial number of a compound is the same as the number of its spectrogram. Also included in table 1 are (a) the conformation (where known) of the corresponding unacetylated glycoside, as determined in the preceding paper in this series [6], and (b) assignments of conformations to the acetylated glycosides. The conformations are indicated by the system devised by Isbell and Tipson [9, 10].

The spectra were measured in the region of 5,000 to 667 cm^−1^ (sodium chloride optics) and in the region of 667 to 250 cm^−1^ (cesium bromide optics). The spectrograms are given together with a discussion of (a) the structure of the compounds and (b) some of the outstanding features of their spectra.

All of the compounds listed in table 1 are fully acetylated glycosides of aldoses, and all have the pyranoid ring. As a common structural feature, all but one of the glycosides have a glycosidic methoxyl group; one has a glycosidic cyclohexyloxy group. The acetylated glycosides differ in regard to one or more of the following structural features: (a) The *α* or *β* anomeric configuration at carbon atom 1; (b) the configurations of the other carbon atoms of the pyranoid ring (including C5 in the hexosides and heptosides); (c) the nature of the substituent, if any, at C5 (including the configuration at C6 of the heptosides); and (d) the nature of the substituent at the C-4 and C-6 hydroxyl groups.

## 3. Reference Aldopymnosides (of Known Conformation) and Their Acetates

In part II of this series, the stable conformation of each of 21 aldopyranosides was deduced from an analysis of the respective infrared absorption spectrum, and was found to be in agreement with the assignment reached from a consideration of instability factors.

In the present study, the crystalline, fully acetylated derivatives of 17 of these aldopyranosides were available (group A). In addition, 7 acetylated aldopyranosides (group B) whose unacetylated relatives had not been available (in the crystalline form) were examined. The spectra of the compounds comprising group A were analyzed, in order to determine if they were explicable on the basis that the acetate of a glycoside has the same conformation as its parent, unacetylated glycoside. The conclusions drawn from this study were then applied to deducing the stable conformation of members of group B.

## 4. Classification of the Acetylated Glycosides Into Configurationally Related Groups

The 24 compounds were classified into three groups; the members of each group have like configurational features.

### 4.1. Acetylated Aldopyranosides of the *xylo* Configuration

The members of this group of acetylated methyl aldopyranosides have the following general formula (I) for the CA conformation.

**Figure:**
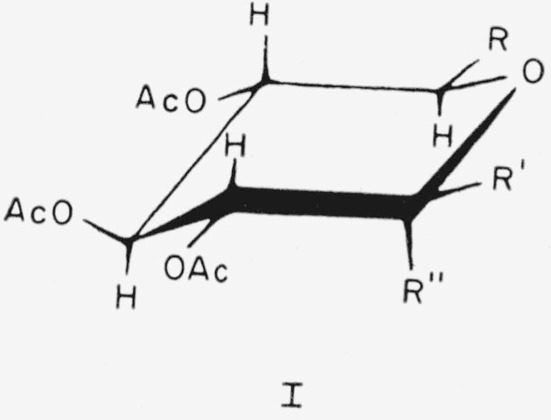


Compounds 1 to 5, in this conformation, have the above general structure, with the following substituents.

1. Methyl *α*-d-xylopyranoside triacetate, R=H; R′ = H; and R″=OCH_3_.
2. Methyl *β*-d-xylopyranoside triacetate, R=H; R′ = OCH_3_; and R″ = H.
3. Methyl *α*-d-glucopyranoside tetraacetate, R = CH_2_OAc; R′=H; and R″ = OCH_3_.
4. Methyl *β*-d-glucopyranoside tetraacetate, R =CH_2_OAc; R′ = OCH_3_; and R″ = H.
5. Methyl *β*-d-glucopyranosyl-(l→6)-*β*-d-glucopyranoside heptaacetate, R=tetra-*O*-acetyl-*β*-d-glucopyranosyl-OH_2_C; R′ = OCH_3_; and R″=H.

Compound 6, if it is the *β* anomer, has the following formulas (II) for the two chair-conformations.

**Figure:**
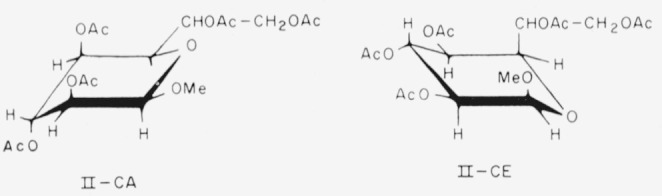

6. Methyl d-*glycero-β*-d-*ido*-heptopyranoside pentaacetate

### 4.2. Acetylated Aldopyranosides of the *lyxo* Configuration

Six of the members of this group of configurationally related compounds have the d-*lyxo* or d-*manno* configuration and the following general formula (III) for the CA conformation.

**Figure:**
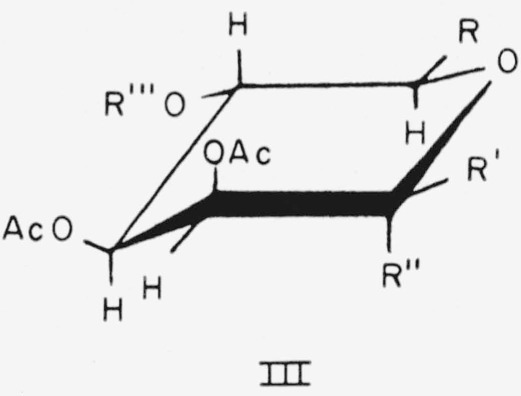


Compounds 7, 8, 11, 12, 14, and 15, in this conformation, have the above general structure, with the following substituents.

7. Methyl *α*-d-lyxopyranoside triacetate, R = H; R′ = H; R″ = OCH_3_; and R‴=Ac.
8. Methyl *β*-d-lyxopyranoside triacetate, R = H; R′ = OCH_3_; R″=H; and R‴=Ac.
11. Methyl *α*-d-mannopyranoside tetraacetate, R = CH_2_OAc; R′=H; R″ = OCH_3_; and R‴ = Ac.
12. Methyl *β*-d-mannopyranoside tetraacetate, R = CH_2_OAc; R′= OCH_3_; R″ = H; and R‴=Ac.
14. Methyl *β*-d-glucopyranosyl-(l→4)-*α*-d-mannopyranoside heptaacetate, R = CH_2_OAc; R′=H; R″=OCH_3_; and R‴=tetra-*O*-acetyl-*β*-d-glucopyranosyl.
15. Methyl *β*-d-glucopyranosyl-(1→4)-*β*-d-mannopyranoside heptaacetate, R = CH_2_OAc; R′ = OCH_3_; R″=H; and R‴ = tetra-*O*-acetyl-*β*-d- glucopyranosyl.

Compounds 9, 10, and 13 have the l-*manno* configuration and the following general formula (IV) for the CA conformation.

**Figure:**
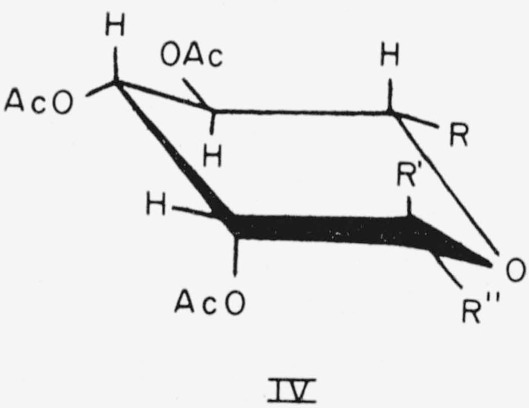

9. Methyl 6-deoxy-*α*-l-mannopyranoside triacetate, R = CH_3_; R′=OCH_3_; and R″=H.
10. Methyl 6-deoxy-*β*-l-mannopyranoside triacetate, R = CH_3_; R′=H; and R″=OCH_3_.
13.

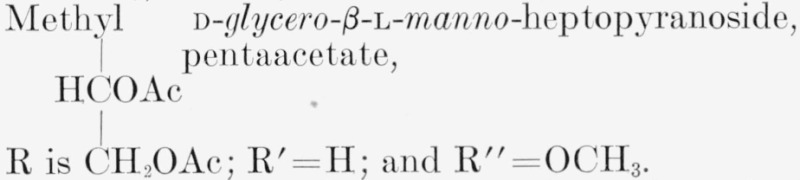

Compounds 16 to 19 have the d-*gulo* configuration and the following general formula (V) for the CA o nfo rmation.

**Figure:**
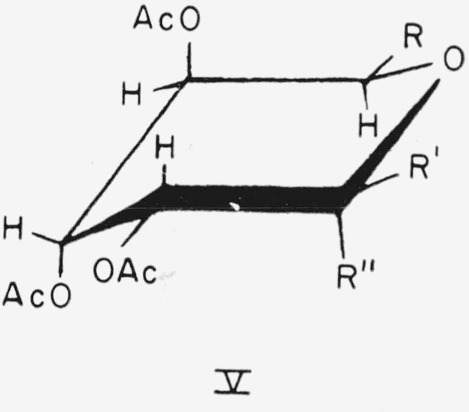

16. Methyl *α*-d-gulopyranoside tetraacetate, R = CH_2_OAc; R′ = H; and R″ = OCH_3_.
17.

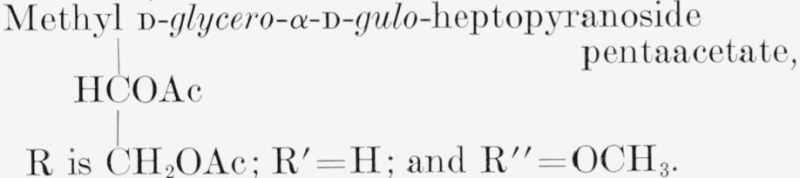
18.

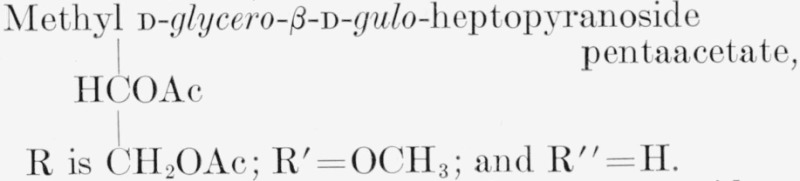
19.

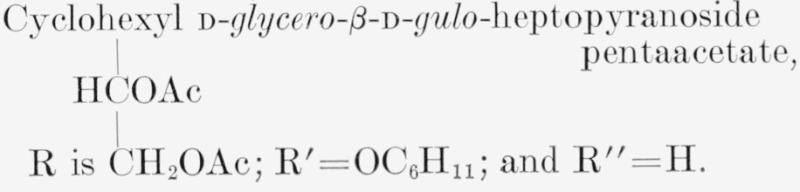

### 4.3. Acetylated Aldopyranosides of the *arabino* Configuration

The CE conformation of compound 20 and the CA conformation of compounds 22 and 23 are depicted in general formula VI.

**Figure:**
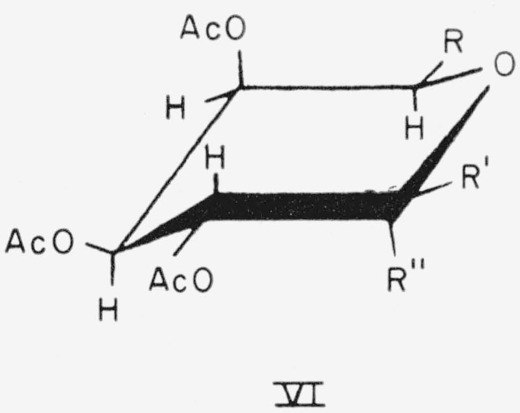

20. Methyl *β*-l-arabinopyranoside triacetate, R = H; R′=H; and R″ = OCH_3_.
22. Methyl *α*-d-galactopyranoside tetraacetate, R = CH_2_OAc; R′=H; and R″ = OCH_3_.
23. Methyl *β*-d-galactopyranoside tetraacetate, R = CH_2_OAc; R′=OCH_3_; and R″ = H.

The CA conformation of compound 21 and the CE conformation of compound 24 are depicted in general formula VII.

**Figure:**
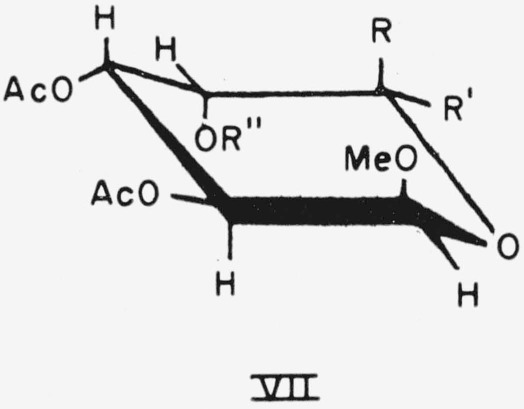

21. Methyl 6-deoxy-*α*-l-galactopyranoside triacetate, R = H; R′ = CH_3_; and R″=Ac.
24. Methyl *β*-d-galactopyranosyl-(1→4)-*β*-d-altropyranoside heptaacetate, R=CH_2_OAc; R′=H; and R″ = tetra-*O*-acetyl-*β*-d-galactopyranosyl.

## 5. Discussion of the Spectra

In the present study, the positions of the various absorption bands for each of 24 acetylated aldopyranosides have been determined; the relative intensities of absorption were not examined in detail. The bands were compiled, and were studied by statistical and comparative methods, as previously described [11].

The conformations of 17 of the corresponding *unacetylated* glycosides had previously been assigned by us [6] from a study of their infrared absorption spectra (see table 1). Assignment was made on the basis of the empirical observation that, for the aldopentopyranosides and for members of any one group- configuration of the 5-*C*-substituted aldopentopyranosides, a change of anomeric disposition was accompanied by a concerted shift of certain groups of absorption bands.

The same kind of examination has now been applied to the spectra of the fully acetylated derivatives of these 17 aldopyranosides. A similar effect was observed, namely, that certain groups of absorption bands show a concerted shift on change of anomeric disposition. Assignments were then made, on the basis of this empirical observation, for 15 of the acetylated derivatives, and were found to be in agreement with those previously made for the unacetylated relatives. Consequently, compounds 3 and 4 were assigned the CA conformation, which had previously been inferred for their parent glycosides [6].

The remaining seven spectra were those of acetylated glycosides whose parent glycosides had received no conformational assignment. Intercomparison of the spectra of four of these acetates (compounds 12 to 15, table 1) with those of related acetates (especially in regard to the characteristic, anomer- differentiating absorption bands) was then under taken. For lack of spectra of related acetates, assignments could not be made for the remaining three compounds (see table 1; compounds 5, 6, and 24).

### 5.1. Absorption Bands Possibly Indicative of the Disposition of Groups at the Anomeric Carbon Atom of the Methyl Tri-*O*-acetyl-aldopentopyranosides

As the starting point in these analyses, we selected methyl tri-*O*-acetyl-*β*-d-xylopyranoside (compound 2) because, if this compound adopts a chair conformation, the reference groups will either be all axial (CE) or all equatorial (CA). Its spectrum was compared with that of its *a* anomer (compound 1), in order to determine the effect, on the spectrum, of changing the anomeric group from equatorial to axial, or vice versa. A comparison was then made with the spectrum of methyl tri-*O*-acetyl-*β*-l-arabinopyranoside (compound 20).

Bands that are essentially the same for these three compounds are given^2^ in table 2. It seemed reasonable to assume tentatively that bands shown by *all* of these acetylated glycosides might be independent of total configuration, whereas those shown by one pair of anomers (*e.g*., compounds 1 and 2) having the same group-configuration might be a reflection, *via* shifting of bands, of an effect of the configuration of that pair.

In table 3 are given the bands shown by *one* anomer (but not by the other) of the methyl tri-*O*- acetyl-d-xylopyranosides and by methyl tri-*O*-acetyl- *β*-l-arabinopyranoside. If the “anomer-differentiating” bands have any relationship to the axial or equatorial disposition of the respective glycosidic methoxyl group, the results in table 3 clearly indicate that compounds 1 and 20 have the same anomeric disposition (that is, both have an equatorial, or both have an axial, methoxyl group), and that the anomeric disposition for compound 2 is different. If the assignment previously made [6] for the unacetylated relative of *any one of these three* acetylated glycosides is extended to its acetate, the conformations of the other two may be deduced from the results in table 3. For example, if the anomeric group of methyl tri-*O*-acetyl-*β*-d-xylopyranoside is equatorial and that of its *α* anomer is axial, the results indicate that the anomeric group of methyl tri-*O*-acetyl-*β*-l-arabinopyranoside is axial. These conclusions are in complete agreement with the assignments previously made [6] for the related unacetylated glycosides.

The corresponding bands of the methyl tri-*O*- acetyl-d-lyxopyranosides (compounds 7 and 8) are also given in table 3. The results suggest that, for each anomer thereof, either (a) a conformation is adopted that is not a chair form and has a quasi, or a different kind of axial or equatorial, anomeric group, or (b) the crystalline material is a mixture of the CA and CE conformations. These conclusions agree with the previous findings for the unacetylated d-lyxopyranosides [6].

### 5.2. Analysis of the Spectra of Groups of Configurationally Related, Acetylated Aldopyrano-sides, Excluding the Acetylated Aldopentopyranosides

As a test of the method of analysis, an examination was made of the spectra of methyl tetra-*O*-acetyl- *α*-d-gulopyranoside (compound 16), the anomers of methyl penta-*O*-acetyl d-*glycero*-d-*gulo*-heptopyranoside (17 and 18), and cyclohexyl penta-*O*-acetyl-d-*glycero-β*-d-*gulo*-heptopynmoside (19), because assignments of anomeric disposition had previously been made [6] for the corresponding unacetylated glycosides. The results are given in table 4, 3 in which the anomer-differentiating bands shown by compounds 17 and 18 are compared with corresponding bands of compound 16 on the one hand and compound 19 on the other. Such bands, shown by two of the compounds (but not by the other two) are listed as follows: Column A, compounds 16 and 17, B, 16 and 18; C, 17 and 19; D, 18 and 19. It may be seen that compound 16 shows about equal similarity to either compound 17 or 18. In contrast, compound 17 has only one such band in common with compound 19, but compounds 18 and 19 have twelve such bands in common. If the assignment previously made for the unacetylated parent is extended to the acetate, for any one of the acetates 17, 18, or 19, the results are in accordance with the assignments previously made for all four parent glycosides. These are, for the anomeric group, as follows: Compound 17, axial; compounds 18 and 19, equatorial; and compound 16, quasi, or a different kind of axial or equatorial disposition, or a mixture of axial and equatorial forms.

As a further check on the results accruing from our method of analysis, a second group of acetylated glycosides (for whose parent glycosides assignments had previously been made [6]) was studied. The results are given in table 5: column A lists three bands shown by methyl tetra-(*O*-acetyl-*β*-d-galactopyranoside (compound 23) but not by compounds 21 and 22; column B lists eleven bands shown by methyl tri-*O*-acetyl-6-deoxy-*α*-l-galactopyranoside (compound 21) and by the *α* anomer, compound 22. Compounds 21 and 23 showed no anomer-differentiating bands in common. Consequently, if the assignment previously made for the unacetylated parent of *any one of these three compounds* is extended to the corresponding acetate, the assignments for the acetates are in harmony with those for the three parent glycosides. For the disposition of the anomeric group, these are as follows: Compounds 21 and 22, axial; and compound 23, equatorial.

All of the foregoing deductions are compatible with the concept that an acetylated glycoside adopts the same conformation as its parent (unacetylated) glycoside. Consequently, because of the agreement between the assignments previously made for the parent glycosides [6] and now made for the acetylated glycosides, the utility of our empirical method for analyzing the spectra (and the validity of the deductions) was apparently established. The spectra of the remaining 12 glycosides were, therefore, examined. No assignment had previously been made as to the stable conformation of compounds 5, 6, 12, 13, 14, 15, and 24.

No assignment could be made for compound 5 (in comparison with compounds 3 and 4) because of the lack of the *α* anomer (with respect to the glycosidic methyl group) of compound 5. However, the anomer-differentiating bands for the anomers of methyl tetra-*O*-acetyl-d-glucopyranoside (compounds 3 and 4) are presented in table 6 for future use.

No assignment could be made for compound 6, because no other acetylated methyl aldopyranosides having the *ido* configuration were available for comparison. Similarly, no assignment could be made for compound 24, because of a lack of acetylated methyl aldopyranosides having the *altro* configuration.

Finally, assignments were sought for compounds 12 to 15; their spectra were compared with those of compounds 9 to 11 (for which we had previously made assignments for the unacetylated glycosides). First of all, the spectra of the anomers of methyl tri-*O*-acetyl-6-deoxy-l-maimopyranoside (compounds 9 and 10) were compared with those of the anomers of methyl tetra-*O*-acetyl-d-mannopyranoside (compounds 11 and 12). The results are given in table 7; column A gives bands shown by compounds 9 and 11 (a anomers) but not by compounds 10 and 12 (*β* anomers), and column B records bands shown by the two *β* anomers but not by the *α* anomers. (Only one discrepancy was noted, namely, that compounds 10 and 11 show a band at 1,168–1,167 cm^−1^ that is absent from the spectra of compounds 9 and 12.) It may be concluded that, assuming the validity of the correlations, if the anomeric group of the *α* anomers is axial, that of the *β* anomers is equatorial, or *vice versa.* If the conformation assigned to the unacetylated parent of any one of three of these four compounds (9, 10, and 11) is accepted for its acetate, the deduced conformations for the other two are in agreement with those for the unacetylated compounds. Furthermore, the results indicate that methyl tetra-*O*-acetyl-*β*-d-mannopyranoside (compound 12) has an equatorial anomeric group.

The anomeric disposition of methyl penta-*O*- acetyl-d-*glycero-β*-l-*manno*-heptopyranoside (13) was now studied by a double comparison—against (a) the anomers of methyl tri-*O*-acetyl-6-deoxy-l-mannopyranoside, and (b) the anomers of methyl tetra-*O*-acetyl-d-mannopyranoside. The results are given in table 8. Column A of table 8 records bands shown by compounds 9 and 13 but not by 10; column B gives bands shown by compound 9 but not by 10 and 13; column C gives bands shown by 10 and 13 but not by 9; column D gives bands shown by 11 and 13 but not by 12; column E gives bands shown by compound 11 but not by 12 and 13; and column I gives bands shown by 12 and 13 but not by 11. It is seen that, whereas the 16 bands in columns B and C indicate similarity between compounds 10 and 13, only two bands in column A suggest similarity of compounds 9 and 13. Similarly, whereas the 18 bands in columns E and F indicate similarity between compounds 12 and 13, only four bands in column D suggest similarity between compounds 11 and 13. Thus, a total of 34 bands indicate relationship of compound 13 to compounds 10 and 12 (as against a total of six bands indicating a relationship of compound 13 to compounds 9 and 11). These results suggest that the anomeric group of compound 13 is equatorial.

To obtain information regarding the disposition of the methoxyl group in the anomers of methyl hepta- *O*-acetyl-4-*O-β*-d-glucopyranosyl-d-marmopyranoside (compounds 14 and 15), the spectra of these compounds were compared with those of the anomers of methyl tetra-*O*-acetyl-d-mannopyranoside (compounds 11 and 12). The results are given in table 9; column A records bands shown by compounds 11 and 14 (but not by compounds 12 and 15) ; column 13 gives the bands shown by compounds 12 and 15 (but not by compounds 11 and 14). If the assignments previously made for compounds 11 and 12 are accepted, these results indicate that the methoxyl group is axial in compound 14 and equatorial in compound 15.

For molecules as complex as those of the acetylated aldopyranosides, many of the observed bands cannot yet be assigned to particular vibrational modes. Thus, in sections 5.1 and 5.2, we have not been concerned with (a) which bands, arising from vibrations localized in a functional group, are relatively independent of the remainder of the molecule, or (b) which bands involve other parts of the molecule. However, in section 5.3, bands possibly attributable to specific functional groups are considered.

### 5.3. Other Absorption Bands

All of the compounds in this study are acetates, and their spectra all show at least one band (C=0 stretching frequency) at 1,764 to 1,736 cm^−1^ (except for compound 5, with a band at 1,773 cm^−1^); at 1,264 to 1,241 cm^−1^ (or at 1,259 to 1,239 cm^−1^); at 1,235 to 1,215 cm^−1^; at 667 to 632 cm^−1^; and at 614 to 585 cm^−1^. All of the spectra show at least one band at 2,994 to 2,941 cm^−1^ (C—H stretching); and at 1,462 to 1,445 cm^−1^ and 1,335 to 1,318 cm^−1^ (C—H bending). All of the spectra show an absorption band at 1,445 to 1,431 cm^−1^ and at 1,379 to 1,368 cm^−1^, presumably caused by deformation of the CH_3_ groups.

**Figure 1:**
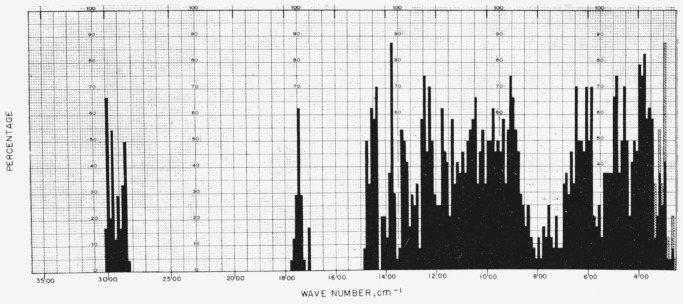
Percentage (of the 24 acetylated glycosides) which showed infrared absorption at the various regions of the infrared spectrum (5,000 to 250 cm^−1^).

**Figure 2:**
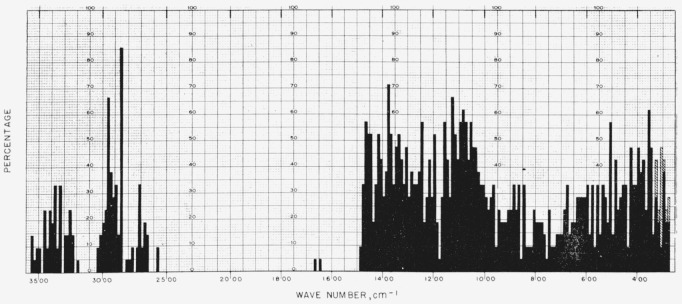
Percentage (of the 21 unacetylated glycosides) which showed infrared absorption at the various regions of the infrared spectrum (5,000 to 250 cm^−1^).

Figure 1 gives the percentage of the 24 compounds in this study that show absorption bands in the various regions of the infrared spectrum; the cross- hatched areas in the region of 333 to 250 cm^−1^ correspond to the region marked, on certain spectra, with dashes. For the range of 5,000 to 2,000 cm^−1^, decrements of 20 cm^−1^ in wavenumber were used; and, for the range of 2,000 to 250 cm^−1^, decrements of 10 cm^−1^. Figure 2 depicts the corresponding “profile” for 21 unacetylated glycosides, compiled from the spectra given in part II of this series [6].

Compound 19, having a cyclohexyloxy group, showed bands at 2,941 and 2,865 cm^−1^, possibly characteristic of —CH_2_— (C—H stretching). It also showed bands at 1,451 and 1,435 cm^−1^, possibly attributable to —CH_2_— (C—H deformation).

All of the acetylated methyl aldopyranosides show a band in the range of 2,882 to 2,833 cm^−1^, excepting compound 4 (band at 2,915 cm^−1^). This band may possibly be attributable to the glycosidic methoxyl group, since Henbest and coworkers [12] have observed that methoxyl groups absorb in the range of 2,832 to 2,819 cm^−1^. All of the acetylated methyl glycopyranosides also show bands at 1,374 to 1,325 cm^−1^, 1,259 to 1,241 cm^−1^, 1,148 to 1,114 cm^−1^, and 1,110 to 1,074 cm^−1^. A band near 1,100 cm^−1^ is characteristic [13] of the methoxyl groups in methoxysteroids.

## 6. Experimental Procedures

### 6.1. Preparation and Purification of the Compounds

The compounds listed in table 1 were prepared by the methods given in the references cited. Most of the compounds were prepared in the course of an earlier study [14] on the structural and configurational relationships of the anomers of the methyl pentopyranosides, hexopyranosides, and heptopyranosides. Each acetate was recrystallized from an appropriate solvent until further recrystallization caused no change in its melting point or optical rotation.

### 6.2. Preparation of the Pellets

Samples for spectrophotometric study were prepared in the solid phase, as pellets consisting of the crystalline acetate suspended in an alkali-metal halide, exactly as previously described [11]. For the range of 5,000 to 667 cm^−1^, a concentration of 0.4 mg of acetate per 100 mg of potassium chloride was used, except for compounds 5 (4 mg/100 mg) and 15 (0.8 mg/100 mg). For the range of 667 to 250 cm^−1^, a concentration of 2 mg of acetate per 100 mg of potassium iodide was used, except for compounds 5 (6 mg/100 mg) and 15 (3 mg/100 mg). Comparisons of intensity of absorption, from one compound to another, can only be true and quantitative where the molar concentration is the same.

### 6.3. Measurement of Infrared Absorption

**Figure 3:**
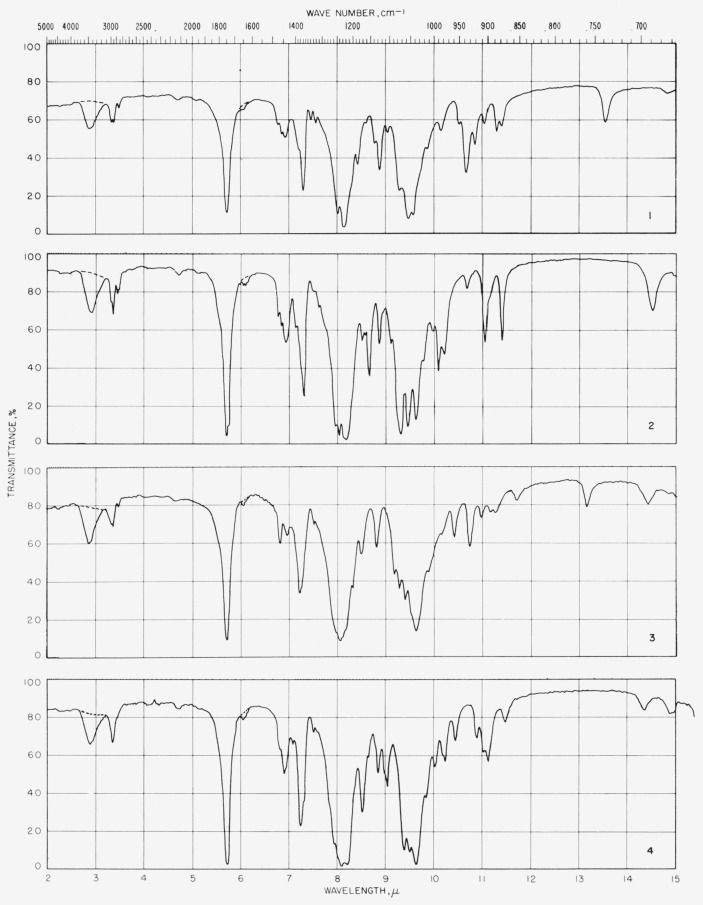

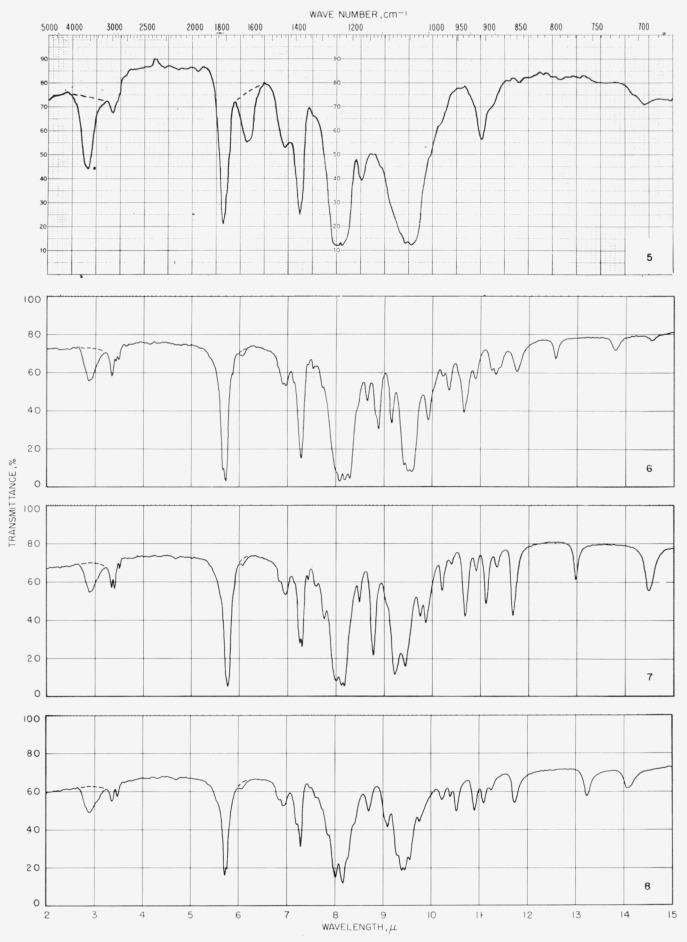

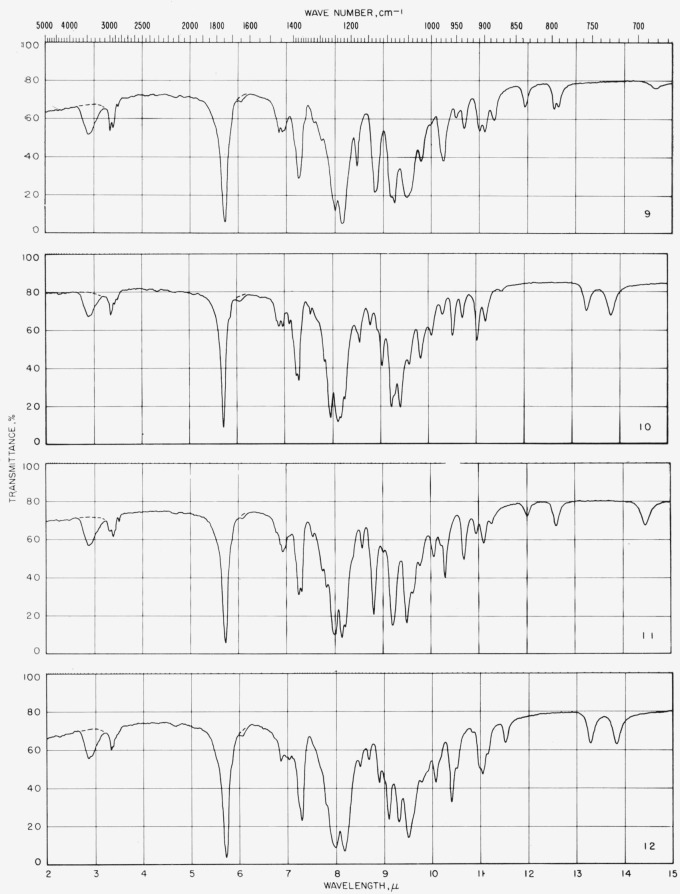

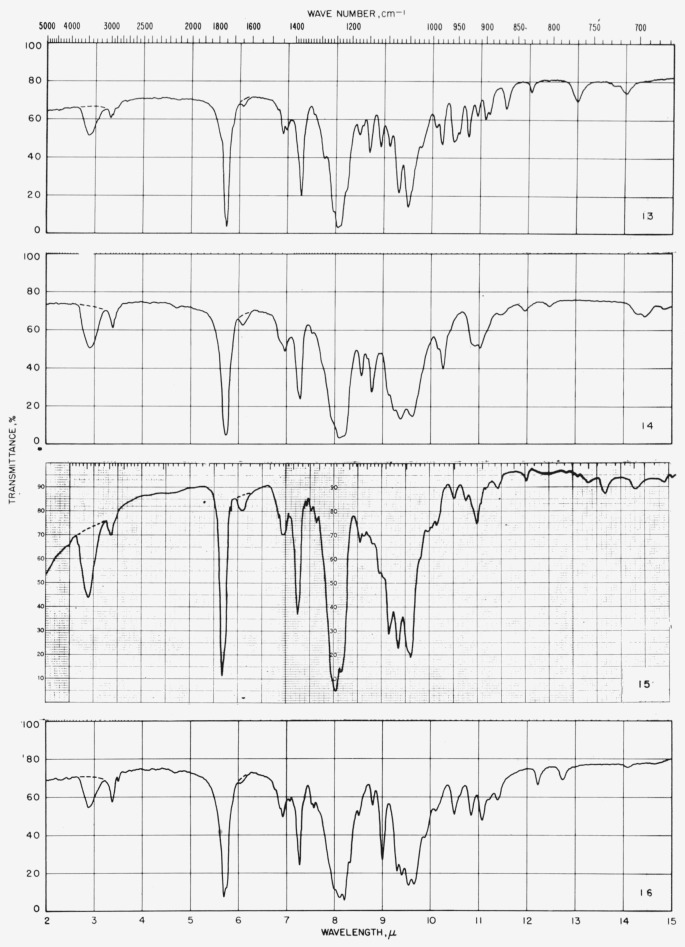

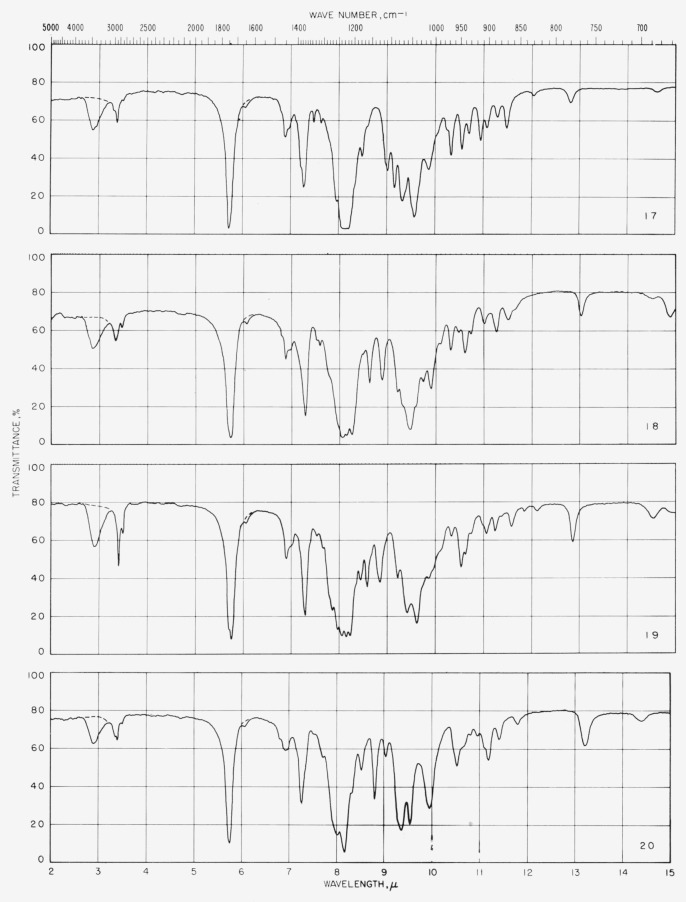

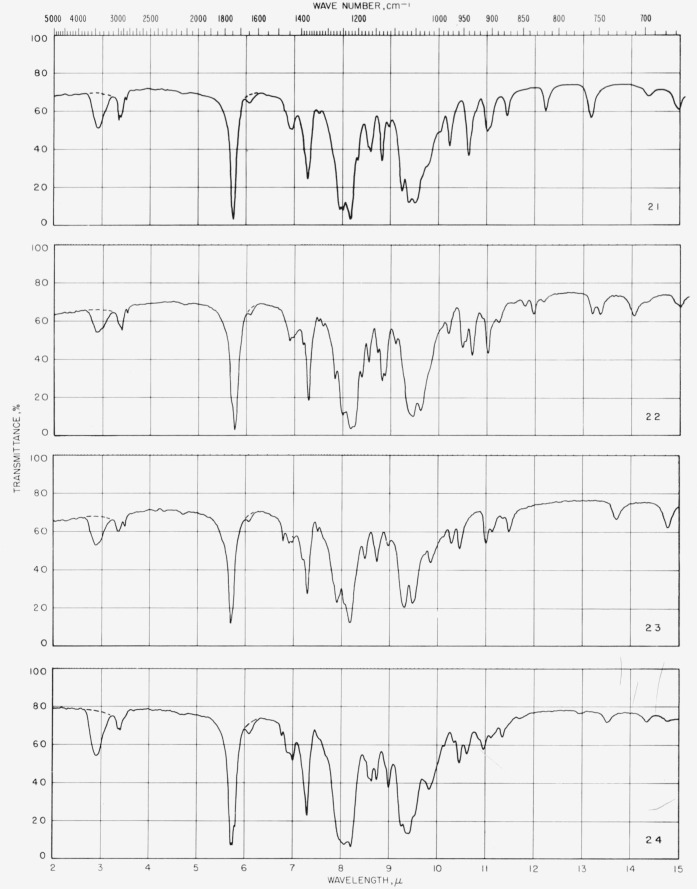
Spectrograms of materials in potassium chloride pellets. **1**, Methyl *α*-d-xylopyranoside triacetate; **2**, methyl *β*-d-xylopyranoside triacetate; **3**, methyl *α*-d-glucopyranoside tetraacetate; **4**, methyl *β*-d-glucopyranoside tetraacetate. **5**, methyl *β*-d-glucopyranosyl-(1→6)-*β*-d-glucopyranoside heptaacetate; **6**, methyl d-*glycero-β*(?)-d-*ido*-heptopyranoside pentaacetate; **7**, methyl *α*-d-lyxopyranoside triacetate; **8**, methyl *β*-d-lyxopyranoside triacetate. **9**, methyl 6-deoxy-*α*-l-mannopyranoside triacetate; **10**, methyl 6-deoxy-*β*-l-mannopyranoside triacetate **11**, methyl *α*-d-mannopyranoside tetraacetate; **12**, methyl *β*-d-mannopyranoside tetraacetate. **13**, methyl d-*glycero-β*-l-*manno*-heptopyranoside pentaacetate; **14**, methyl *β*-d-glucopyranosyl-(1→4)-*α*-d-mannopyranoside heptaacetate; **15**, methyl *β*-d-glucopyranosyl-(1→4)-*β*-d-mannopyranoside heptaacetate; **16**, methyl *α*-d-gulopyranoside tetraacetate. **17**, methyl d-*glycero-α*-d-*gulo*-heptopyranoside pentaacetate; **18**, methyl d-*glycero-β*-d-*gulo*-heptopyranoside pentaacetate: **19**, cyclohexyl d-*glycero-β*-d-*gulo*-heptopyranoside pentaacetate; **20**, methyl *β*-l-arabinopyranoside triacetate. **21**, methyl 6-deoxy-*α*-l-galactopyranoside triacetate; **22**, methyl *α*-d-galactopyranoside tetraacetate; **23**, methyl *β*-d-galactopyranoside tetraacetate; 24, methyl *β*-d-galactopyranosyl-(1→4)-*β*-d-altropyranoside heptaacetate.

**Figure 4:**
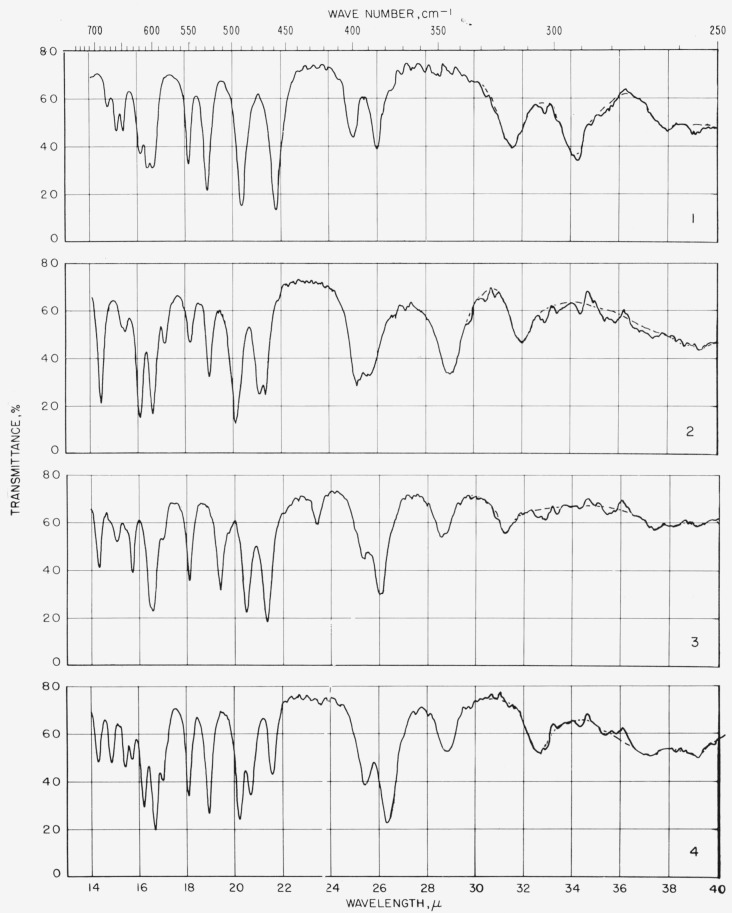

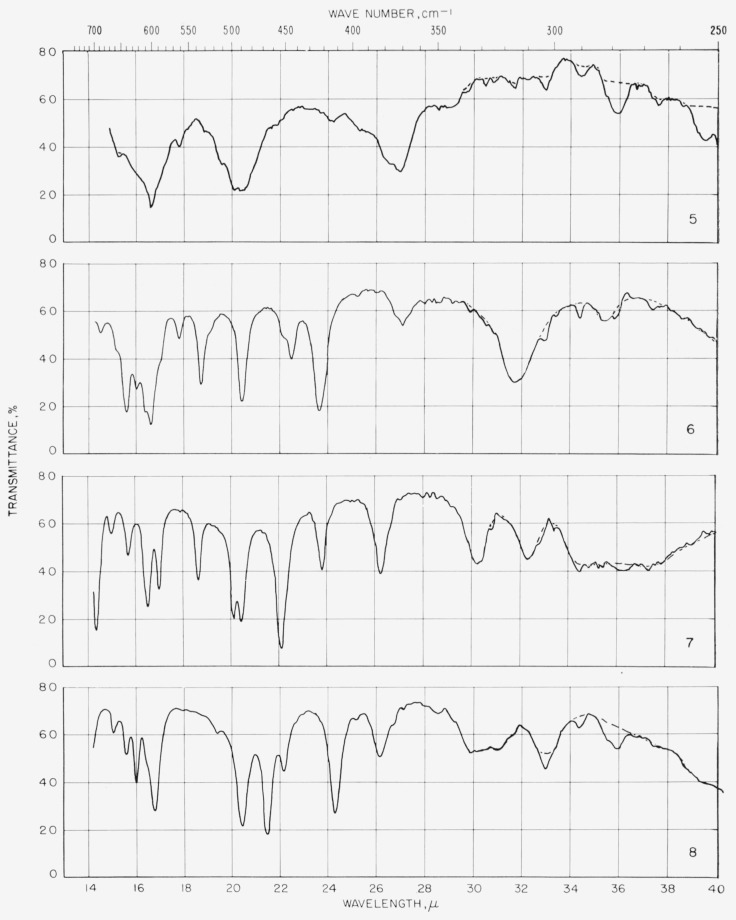

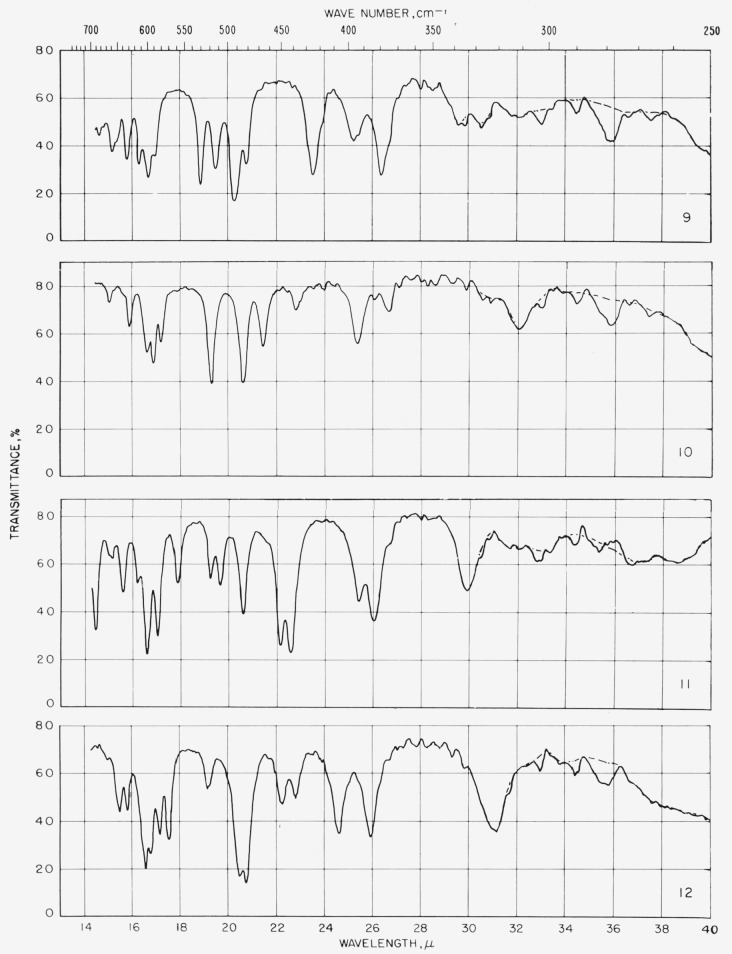

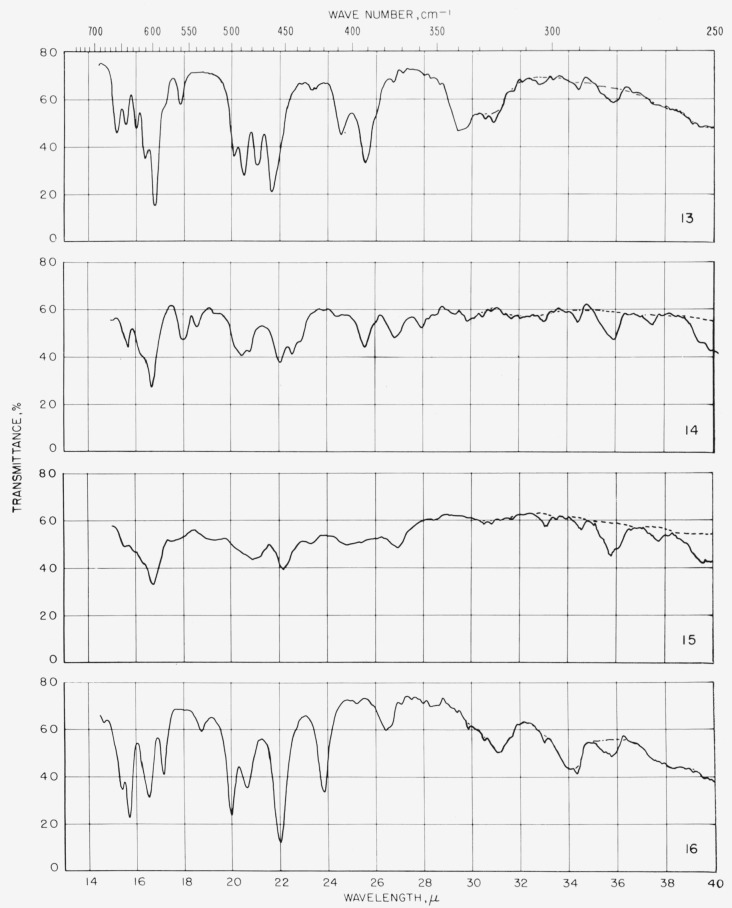

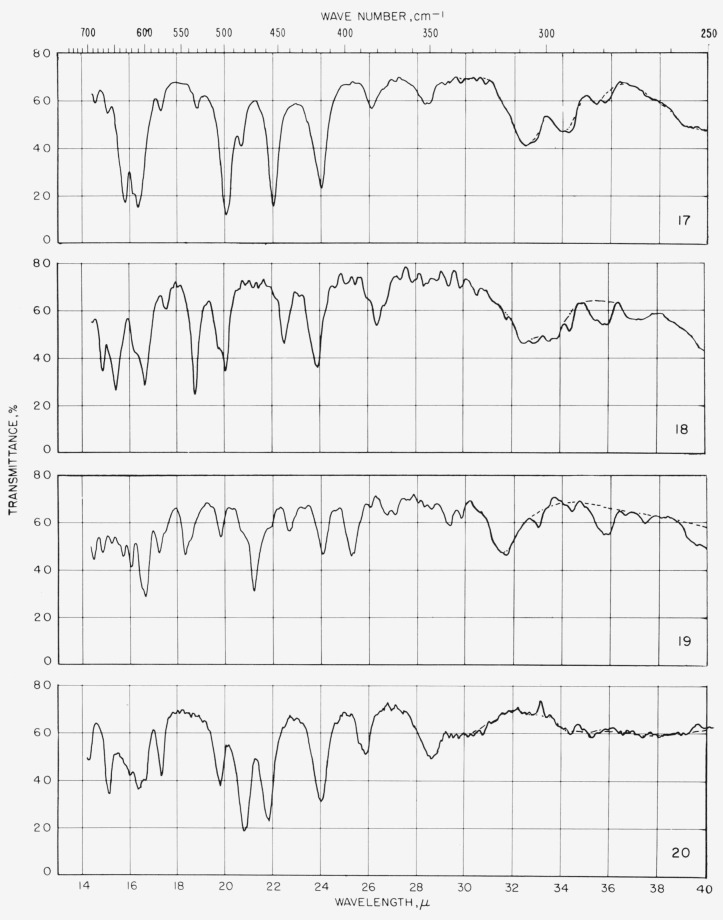

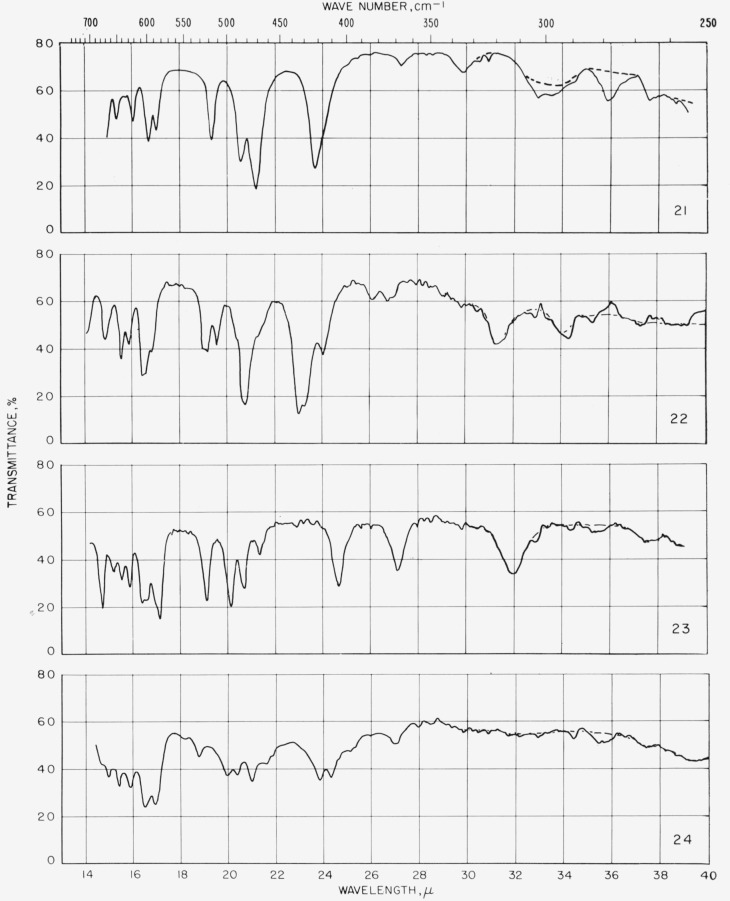
Spectrograms of materials in potassium iodide pellets. **1**, Methyl *α*-d-xylopyranoside triacetate; **2**, methyl *β*-d-xylopyranoside triacetate; **3**, methyl *α*-d-glucopyranoside tetraacetate; **4**, methyl *β*-d-glucopyranoside tetraacetate. **5**, methyl *β*-d-glucopyranosyl-(1→6)-*β*-d-glucopyranoside heptaacetate; **6**, methyl d-glycero-*β*(?)-d-ido-heptopyranoside pentaacetate; **7**, methyl *α*-d-lyxopyranoside triacetate; **8**, methyl *β*-d-lyxopyranoside triacetate. **9**, methyl 6-deoxy-*α*-l-mannopyranoside triacetate; **10**, methyl 6-deoxy-*β*-l-mannopyranoside triacetate; **11**, methyl *α*-d-mannopyranoside tetraacetate; **12**, methyl *β*-d-mannopyranoside tetraacetate. **13**, methyl d-glycero-*β*-l-manno-heptopyranoside pentaacetate; **14**, methyl *β*-d-glucopyranosyl-(1→4)-*α*-d-mannopyranoside heptaacetate; **15**, methyl *β*-d-glucopyranosyl-(1→4)-*β*-d-mannopyranoside heptaacetate; **16**, methyl *α*-d-gulopyranoside tetraacetate. **17**, methyl d-glycero-*α*-d-gulo-heptopyranoside pentaacetate; **18**, methyl d-glycero-*β*-d-gulo-heptopyranoside pentaacetate; **19**, cyclohexyl d-glycero-*β*-d-gulo-heptopyranoside pentaacetate; **20**, methyl *β*-l-arabinopyranoside triacetate. **21**, methyl 6-deoxy-*α*-l-galactopyranoside triacetate; **22**, methyl *α*-d-galactopyranoside tetraacetate; **23**, methyl *β*-d-galactopyranoside tetraacetate; 24, methyl *β*-d-galactopyranosyl-(1→4)-*β*-d-altropyranoside heptaacetate.

The spectrograms are shown in figures 3 and 4. Those in figure 3 for compounds 5 and 15 were recorded with a Beckman Model IR4 (double-beam) spectrophotometer equipped with prisms of sodium chloride. The others were recorded with a Perkin- Elmer Model 21 (double-beam) spectrophotometer equipped with a prism of sodium chloride (for the range of 5,000 to 667 cm^−1^) and of cesium bromide (for the range of 667 to 250 cm^−1^), as previously described [11].

Some absorption attributable to water (in the compound, the alkali halide, or both) was observed at 3,448 and 1,639 cm^−1^ and, attributable to atmospheric water vapor, in the far-infrared curves. These regions are drawn on the spectrograms with dashed lines which merely indicate uncertainty and are not to be interpreted quantitatively.

### 6.4. Spectra Measured Under Different Conditions

The spectra of 13 of the acetylated aldopyranosides (compounds 1 to 4, 7, 9 to 12, 18, 20, 22, and 23) had previously been measured [3] in carbon tetrachloride and in either carbon disulfide, dioxane, or chloroform. As has previously been mentioned, the infrared absorption spectra of crystalline materials show more bands than the spectra of the same compounds in solution. As a result, a larger number of bands were available for correlations than in the previous study [3].

## Figures and Tables

**Table 1:** Compounds measured, stable conformations, and index to spectrograms

Code^a^	Compound	Reference	Stable conformation^b^			Spectrogram
			Assignment for unacetylated glycoside^c^	Present assignment^d^	Anomeric disposition^d^	
						
12.11111(2,3,4)21	Methyl *α*-d-xylopyranoside, triacetate	1	CA	CA	*a*	1
12.11211(2,3,4)21	Methyl *β*-d-xylopyranoside, triacetate	2, 3	CA	CA	*e*	2
12.21111(2,3,4,6)21	Methyl *α*-d-glucopyranoside, tetraacetate	4 to 6	CA	CA	*a*	3
12.21211(2,3,4,6)21	Methyl *β*-d-glucopyranoside, tetraacetate	4, 6	CA	CA	*e*	4
22.21?(2,3,4,6)21^*^(1-6).21?11(2,3,4)21	Methyl *β*-d-glucopyranosyl-(l→6)-*β*-d-glucopyranoside, heptaacetate.^e^	7	……….	……….	……….	5
12.35?11(2,3,4,6,7)21	Methyl d-*glycero-β*(?)-d-*ido*-heptopyranoside, pentaacetate.	8	……….	……….	……….	6
						
12.12511(2,3,4)21	Methyl *α*-d-lyxopyranoside, triacetate	9,10	CA+CE; non-chair.	CA+CE; non-chair.	*a+e; a,e,.* or *q.*	7
12.12511(2,3,4)21	Methyl *β*-d-lyxopyranoside, triacetate	11	CA+CE; non-chair.	CA+CE; non-chair.	*a+e; a,e, or q.*	8
12.22111(2,3,4)21(6)80	Methyl 6-deoxy-*α*-l-mannopyranoside, triacetate	12,13	CA	CA	*a*	9
12.22211(2,3,4)21(6)80	Methyl 6-deoxy-*β*-l-mannopyranoside, triacetate	12	CA	CA	*e*	10
12.22111(2,3,4,6)21	Methyl *α*-d-mannopyranoside, tetraacetate	14,15	CA	CA	*a*	11
12.22211(2,3,4,6)21	Methyl *β*-d-mannopyranoside, tetraacetate	14,15	……….	CA	*e*	12
12.42211(2,3,4,6,7)21	Methyl d-*glycero-β*-l-*manno*-heptopyranoside, pentaacetate.	11	……….	CA	*e*	13
22.21?(2,3,4,6)21^*^(1-4).22111(2,3,6)21	Methyl *β*-d-glucopyranosyl-(1→4)-*α*-d-mannopyranoside, heptaacetate.	16,17	……….	CA	*a*	14
22.21?(2,3,4,6)21^*^(1-4).22211(2,3,6)21	Methyl *β*-d-glucopyranosyl-(1→4)-*β*-d-mannopyranoside, heptaacetate.	16	……….	CA	*e*	15
12.26511(2,3,4,6)21	Methyl *α*-d-gulopyranoside, tetraacetate	18	CA+CE; non-chair.	CA+CE; non-chair.	*a+e; a,e*, or *q.*	16
12.36111(2,3,4,6,7)21	Methyl d-*glycero-α*-d-*gulo*-heptopyranoside, pentaacetate.	11	CA	CA	*a*	17
12.36211(2,3,4,6,7)21	Methyl d-*glycero-β*-d-*gulo*-heptopyranoside, pentaacetate.	19	CA	CA	*e*	18
12.36213(2,3,4,6,7)21	Cyclohexyl d-*glycero-β*-d-*gulo*-heptopyranoside, pentaacetate.	20	CA	CA	*e*	19
						
12.13311(2,3,4)21	Methyl *β*-l-arabinopyranoside, triacetate	21	CE	CE	*a*	20
12.23111(2,3,4)21(6)80	Methyl 6-deoxy-*α*-l-galactopyranoside, triacetate	22	CA	CA	*a*	21
12.23111(2,3,4,6)21	Methyl *α*-d-galactopyranoside, tetraacetate	23, 24	CA	CA	*a*	22
12.23211(2,3,4,6)21	Methyl *β*-d-galactopyranoside, tetraacetate	6, 24	CA	CA	*e*	23
22.23?(2,3,4,6)21^*^(1-4).27?11(2,3,6)21	Methyl *β*-d-galactopyranosyl-(l→4)-*β*-d-altropyranoside, heptaacetate.	25	……….	……….	……….	24

a The third figure after the point was inserted after the present conclusions as to conformation had been reached.

b Named by the system of H. S. Isbell and R. S. Tipson, Science **130**, 793 (1959); J. Research NBS **64A**, 171 (1960).

c Assignment made by R. S. Tipson and H. S. Isbell, J. Research NBS **64A**, 239 (1960).

d After accepting several of the assignments for the unacetylated glycosides (see text).

e A sample of the original material, prepared by J. M. Johnson in November 1916, was kindly presented by N. K. Richtmyer.

References for table 1

1. C. S. Hudson and J. K. Dale, *J. Am. Chem. Soc.* **40**, 997 (1918).

2. C. S. Hudson and J. M. Johnson, *J. Am. Chem. Soc*. **37**, 2748 (1915).

3. J. K. Dale, *J. Am. Chem. Soc.* **37**, 2746 (1915).

4. C. S. Hudson and J. K. Dale, *J. Am. Chem. Soc*. **37**, 1264 (1915).

5. E. Fischer and E. F. Armstrong, *Ber. deut. chem. Ges*. **34**, 2890 (1901).

6. W. Koenigs and E. Knorr, *Ber. deut. chem. Ges*. **34**, 957 (1901).

7. C. S. Hudson and J. M. Johnson, *J. Am. Chem. Soc*. **39**, 1272 (1917).

8. H. L. Frush and H. S. Isbell, *J. Research NBS* **35**, 111 (1945) RP1663.

9. P. A. Levene and M. L. Wolfrom, *J. Biol. Chem*. **78**, 525 (1928): **79**, 471 (1928).

10. F. P. Phelps and C. S. Hudson, *J. Am. Chem. Soc*. **50**, 2049 (1928).

11. H. S. Isbell and H. L. Frush, *J. Research NBS* **24**, 125 (1940) RP1274.

12. E. Fischer, M. Bergmann, and A. Rabe, *Ber. deut. chem. Ges*. **53**, 2362 (1920).

13. M. Bergmann and H. Schotte, *Ber. deut. chem. Ges*. **54**, 1569 (1921).

14. J. K. Dale, *J. Am. Chem. Soc*. **46**, 1048 (1924).

15. T. L. Harris, E. L. Hirst, and C. E. Wood, *J. Chem. Soc*. **1932**, 2108.

16. H. S. Isbell, *BS J. Research* **7**, 1115 (1931) RP392.

17. W. N. Haworth, E. L. Hirst, H. R. L. Streight, H. A. Thomas, and J. I. Webb, *J. Chem. Soc*. **1930**, 2636.

18. H. S. Isbell, *BS J. Research* **8**, 1 (1932) RP396.

19. W. N. Haworth, E. L. Hirst, and M. Stacey, *J. Chem. Soc*. **1931**, 2864.

20. E. Glaser and N. Zuckermann, *Z. physiol. Chem*. **166**, 103 (1927).

21. C. S. Hudson and J. K. Dale, *J. Am. Chem. Soc*. **40**, 992 (1918).

22. J. Minsaas, *Rec. trav. chim*. **56**, 623 (1937).

23. F. Micheel and O. Littmann, *Liebigs Ann. Chem*. **466**, 115 (1928).

24. J. K. Dale and C. S. Hudson, *J. Am. Chem*. Soc. **52**, 2534 (1930).

25. H. L. Frush and H. S. Isbell, *J. Research NBS* **27**, 413 (1941) RP1429.

**Table 2:** Bands (cm^−1^) shown by both anomers of methyl tri-*O*-acetyl-*d*-xylopyranoside (compounds 1 and 2) and by methyl tri-*O*-acetyl-*β*-*l*-arabinopyranoside (20), and positionally corresponding bands of the methyl tri-*O*-acelyl-*d*-lyxopyranosides (7 and 8)

Methyl tri-*O*-acetyl-d-xylopyranosides		Methyl tri-*O*-acetyl-*β* -l-arabinopyranoside	Methyltri-*O*-acetyl-d-lyxopyranosides	
1	2	20	7	8

Possibly non-configurational bands				

1745	1742	1739	1736	1733
1456	1462	1453	1462	1456
1441	1443	1445, 1437	1437	1439
1383	1376	1377	1377	1385
1339	1332	1333	1348	1333
1294	1287	1299	1290	1272
1247	1244	1247	1250	1247
1225	1221	1224	1224	1222
1185	1175	1172	1179	1190
1105	1100	1107	1104	1099
a1075	a1075	a1068	1085	1074
885	899	895	899	889
674	689	695	690	710
586	585	578	590	596
491	499	506	498, 491	491
459	458	459, 447	453	453
401	399	407	408(?)	399
385	392	387	382	383
376	375	378(?)	376	376
344	346	345(?)	332	336(?), 332(?)
292	291(?)	291(?)	291(?)	291(?)

Bands possibly affected by configuration and conformation				

1473	1477	1468	……………	……………
1368	1370	……………	1370	1370
1321	1316	……………	1318	1311
1161	1167	……………	……………	……………
b1138	1129	b1136	b1140	……………
1124	1119	……………	……………	……………
a1055	b1058	……………	1060	1064, 1057
1044	1040	a1048	……………	1045
1031	1024	……………	1027	1024
1013	1003	1006	1014	……………
984	980	……………	980	977
……………	……………	……………	963	962
a936	936	938	936	……………
……………	……………	913	917	917
904	906	902	……………	901
877	880	877	882	……………
……………	……………	848	856	851
648	647	……………	638	642
619	621	626	……………	625
600	601	601	……………	……………
551	550	……………	……………	……………
528	527	……………	538	517
479	475, 470	482	……………	466
……………	……………	418	421	413
371	370	371(?)	……………	……………
317	313	……………	310	……………

a These bands were mentioned by R. L. Whistler and L. R. House [4].

b These bands were mentioned by H. S. Isbell and coworkers [3].

**Table 3:** Bands shown by only one anomer of the methyl tri-*O*-acetyl-*d*-xylopyranosides (compounds 1 and 2) and by methyl tri-*O*-acetyl-*β*-*l*-arabinopyranoside (20), compared with bands for both anomers of methyl tri-*O*-acetyl-*d*-lyxopyranoside (7 and 8)

1	20	2	7	8
				
2933	2941	……….	2941	……….
2841	2849	……….	2849	……….
a1205	a1202	……….	………	1209
949	951	………..	……….	949
921	926	………	………..	………..
738	757	………..	771	755
659	660	………..	668	665
607	612	……….	606	608
366	365(?)	………..	………..	………..
358	359	…………	………	…………
350	350	…………	………..	351(?)
263	284(?)	………….	………..	…………
………..	……….	3012	………….	…………
………..	………..	2967	………….	2967
………..	………..	2890	2899	……………
………..	……….	2874	…………	2865
………..	………..	1754	……….	1745
…………	………..	1403	1399	…………
………..	…………	1256	…………	………..
……….	…………	………..	1233	…………
………..	………..	1155	…………	1148
…………	…………	992	………………	……………

a See footnote b of table 2.

**Table 4:** Comparison^a^ of absorption bands (cm^−1^) shown by methyl tetra-*O*-acetyl-*α*-*d*-gulopyranoside (compound 16), the anomers of methyl penla-*O*-acetyl-*d*-glycero-*d*-gulo-heptopyranoside (17 and 18), and cyclohexyl penta-*O*-acetyl-*d*-glycero-*β*-*d*-gulo-heptopyranoside (19)

A		B		C		D	
16(*α*)	17(*α*)	16(*α*)	18(*β*)	17(*α*)	19(*β*)	18(*β*)	19(*β*)
							
2841	2849	1736	1745	831	825	2882	2865
1414	1385	943	943			1745	1739
1110	1110	650	649			1282	1274
818	831					1241	1242
						943	942
						858	864
						649	645
						508	506
						472	473
						459	459
						445	443
						341	341

a Key: A. Bands shown by compounds 16 and 17, but not by 18. B. Bands shown by compounds 16 and 18, but not by 17. C. Bands shown by compounds 17 and 19, but not by 18. D. Bands shown by compounds 18 and 19, but not by 17.

**Table 5:** Comparison a of absorption bands (cm^−1^) shown by the anomers of methyl tetra-*O*-acetyl-*d*-galactopyranoside (compounds 22 and 23) and by methyl tri-*O*-acelyl-6-deoxy-*α*-*l*-galaclopyranoside (21)

A	B	
23 (*β*)	21(*α*)	22(*α*)
		
1014	2933	2941
b880	1215	1217
611	1200	c1190
	1133	c1134
	1049	1041
	941	b949
	928	b936
	817	b821
	759	b758
	696	712
	487	490

a Key: A. Bands shown by compound 23, but not by compounds 21 and 22. B. Bands shown by compounds 21 and 22, but not by 23.

b These bands were mentioned by S. A. Barker and coworkers [5].

c See footnotes a and b to table 2.

**Table 6:** Bands (cm^−1^) differentiating between the anomers of methyl ietra-*O*-acetyl-*d*-glucopyranoside (compounds 3 and 4)

3(*α*)	4(*β*)
	
2865	…………
1241	…………
1078	…………
933	…………
888	…………
761	…………
663	…………
516	…………
321	…………
…………	2915
…………	1318
…………	a918
…………	a873
…………	537(?)
…………	529

a See footnote b to table 5.

**Table 7:** Comparison a of absorption bands (cm^−1^) shown by the methyl tri-*O*-acetyl-6-deoxy-*l*-mannopyranosides (compounds 9 and 10) and by the methyl tetra-*O*-acelyl-*d*-mannopyranosides (11 and 12)

A		B	
9(*α*)	11(*α*)	10(*β*)	12(*β*)
			
1443	1443	1410	1404
b884	b886	1076	1074
b 837	b 832	867	b 867
b797, b791	b792	752	b752
685	690	724	723
614	616	412,406	408
514	510		

a Key: A. Bands shown by compounds 9 and 11, but not by compounds 10 and 12. B. Bands shown by compounds 10 and 12, but not by compounds 9 and 11.

b See footnote b to table 5.

**Table 8:** Comparison a of absorption bands (cm^−1^) shown by the methyl tri-*O*-acetyl-6-deoxy-*l*-mannopyranosides (compounds 9 and 10), the methyl tetra-*O*-acetyl-*d*-mannopyranosides (11 and 12), and methyl penta-*O*-acetyl-*d*-glycero-*β*-*l-manno*-heptopyrano side (13)

A		B	C		D		E	F	
9(*α*)	13(*β*)	9(*α*)	10(*β*)	13(*β*)	11(*α*)	13(*β*)	11(*α*)	12(*β*)	13(*β*)
									
b837	829	1443	1410	1410(?)	1227	1232	1443	1404	1410(?)
495	499	b884	1168	1167	1167	1167	1086	1151	1147
		b797, b791	1076	1073	b832	829	1040	1074	1073
		685	894	894	465(?)	462	b886	b952	947
		614	867	867			b792	b867	867
		514	752	768			690	b752	768
			724	725, 713			510	723	725, 713
			487	489				595	596
			447	447				484	476
			412, 406	409				408	409
								342(?)	341

a Key: A. Bands shown by compounds 9 and 13, but not by 10. B. Bands shown by compound 9, but not by 10 and 13. C. Bands shown by compounds 10 and 13, but not by 9. D. Bands shown by compounds 11 and 13, but not by 12. E. Bands shown by compound 11, but not by 12 and 13. F. Bands shown by compounds 12 and 13, but not by 11.

b See footnote b to table 5.

**Table 9:** Comparison^a^ of absorption bands (cm^−1^) shown by the methyl tetra-*O*-acetyl-*d*-mannopyranosides (compounds 11 and 12) and by the methyl hepla-*O*-acetyl-4-*O*-*β*-*d*-glucopyranosyl-*d*-mannopyr ano sides (14 and 15)

A		B	
11(*α*)	14(*α*)	12(*β*)	15(*β*)
			
1086	1083	1010	1003
978	976	b952	952
b792	803	b904	904
690	693	b752	750
616	614	723	731
559	557	570	567

a Key: A. Bands shown by compounds 11 and 14, but not by 12 and 15. B. Bands shown by compounds 12 and 15, but not by 11 and 14.

b See footnote b to table 5.

## Footnotes

1 Figures in brackets indicate the literature references at the end of this paper. The references for table 1 are given at the end of the table.

2 For comparison, bands (in about the same positions) shown by either or both anomers of methyl *tri-O-acetyl-d-lyxopyranoside* are also given in table 2.

3 To conserve space, bands which do not differentiate between anomers havebeen omitted from tables 4 to 9.
